# Racial/Ethnic Disparities in Pregnancy and Prenatal Exposure to Endocrine-Disrupting Chemicals Commonly Used in Personal Care Products

**DOI:** 10.1007/s40572-021-00317-5

**Published:** 2021-05-27

**Authors:** Marissa Chan, Carol Mita, Andrea Bellavia, Michaiah Parker, Tamarra James-Todd

**Affiliations:** 1 Department of Environmental Health, Harvard T.H. Chan School of Public Health, 665 Huntington Ave., Bldg. 1, 14th Floor, Boston, MA 02115 USA; 2 Countway Library, Harvard Medical School, Boston, MA 02115 USA; 3 Department of Epidemiology, Harvard School of Public Health, Boston, MA 02115 USA; 4 Division of Women’s Health, Department of Medicine, Connors Center for Women’s Health and Gender Biology, Brigham and Women’s Hospital and Harvard Medical School, Boston, MA 02120 USA

**Keywords:** Race, Ethnicity, Endocrine disruptors, Pregnancy, Prenatal exposure

## Abstract

**Purpose of Review:**

Endocrine-disrupting chemical (EDC) exposure during pregnancy is linked to adverse maternal and child health outcomes that are racially/ethnically disparate. Personal care products (PCP) are one source of EDCs where differences in racial/ethnic patterns of use exist. We assessed the literature for racial/ethnic disparities in pregnancy and prenatal PCP chemical exposures.

**Recent Findings:**

Only 3 studies explicitly examined racial/ethnic disparities in pregnancy and prenatal exposure to PCP-associated EDCs. Fifty-three articles from 12 cohorts presented EDC concentrations stratified by race/ethnicity or among homogenous US minority populations. Studies reported on phthalates and phenols. Higher phthalate metabolites and paraben concentrations were observed for pregnant non-Hispanic Black and Hispanic women. Higher concentrations of benzophenone-3 were observed in non-Hispanic White women; results were inconsistent for triclosan.

**Summary:**

This review highlights need for future research examining pregnancy and prenatal PCP-associated EDCs disparities to understand and reduce racial/ethnic disparities in maternal and child health.

**Supplementary Information:**

The online version contains supplementary material available at 10.1007/s40572-021-00317-5.

## Introduction

Racial/ethnic disparities in early-life health outcomes have been well-documented, with notable disparities including preterm birth [1–3], low birth weight [1–3], early onset of puberty [4, 5], and childhood asthma [6, 7]. Many of these conditions affect non-Hispanic Black, Hispanic, and Asian populations to a greater extent compared to non-Hispanic Whites [1, 8, 9]. While a variety of social and lifestyle factors are at play, recent work suggests that endocrine-disrupting chemicals (EDCs) during pregnancy and the prenatal period may also play a role in these disparities [10]. Specifically, non-Hispanic Black women have higher concentrations of many EDCs that are linked to these adverse health outcomes [11–15].

While there are numerous sources of EDCs, including food, clothing, and other consumer products, many of the classes of EDCs that are racially/ethnically disparate are found in personal care products (PCPs), where culturally driven patterns of product use exist [16]. Personal care product use during pregnancy is an important and understudied source of prenatal exposure to EDCs, with implications for later-life maternal and child health [17, 18]. Although research documents exposure to EDC-associated personal care products during pregnancy and the prenatal period [16, 19, 20], as well as associations of EDC concentrations with adverse maternal and child health outcomes, few studies have examined the racial/ethnic patterns of use that may contribute to these higher EDC exposures and their associations with disparate maternal and child health outcomes [10]. Of interest, PCPs may be a possible modifiable factor that could provide opportunities for intervention to reduce racial/ethnic disparities in EDC exposure.

### Pregnancy and Prenatal EDC Exposures as Contributors to Racial/Ethnic Disparities in Maternal and Child Health Outcomes

The Developmental Origins of Health and Disease theory hypothesizes that early life environmental exposures, particularly those occurring during the prenatal period, can have lasting effects on later life health. Thus, when considering EDC exposure, the prenatal period can be a critical window of exposure [21, 22]. Indeed, studies show that higher exposure to EDCs during the prenatal period, including those commonly found in personal care products, has been linked to a number of adverse child health outcomes including low birth weight [23] and preterm birth [13]. For example, data from the racially/ethnically diverse LIFE Study showed that moderately high concentrations of the metabolite monoethyl phthalate (MEP) were associated with over a 200-g decrease in birth weight (*β* = −200.2; 95% CI: −386.9, −13.4) [23]. Interestingly, non-Hispanic Black women, as well as women from certain Asian subgroups, are 2-times more likely to have a low birth weight infant, suggesting the need to determine whether differences in phthalate exposure could contribute to racial/ethnic disparities in birth weight [1–3].

Studies also show associations with decreased gestational age and higher exposure to certain phthalates. For example, in a study of the Puerto Rico Testsite for Exploring Contamination Threats (PROTECT) cohort evaluating 1090 Puerto Rican women, each interquartile range increase in average pregnancy monobutyl phthalate (MBP) concentrations was associated with a 42% increased odds of preterm birth (odds ratio=1.42 95%CI 1.07, 1.88) [24]. With non-Hispanic Black women having a 50% higher risk of preterm birth, this association may suggest the need to evaluate whether EDCs contribute to the striking disparity that leads to adverse infant and child health outcomes. Other adverse health outcomes linked to higher exposure to EDCs exists, such as neurobehavioral, asthma, and allergic disease outcomes [20, 25, 26], conditions that are more prevalent in non-Hispanic Black and Hispanic populations. While these disparities are known, and studies show associations between EDCs and these health outcomes, few studies have evaluated whether EDCs could be a modifiable risk factor for these racially/ethnically disparate maternal and child health outcomes.

### Racial/Ethnic Disparities in EDCs Commonly Found in Personal Care Products

Identifying sources of EDC exposure known to be racially/ethnically disparate may provide critical information for risk reduction in vulnerable populations. Personal care products are an important source of many EDCs that are racially/ethnically disparate; these products have been hypothesized to be key for environmental health disparities [16, 27–29]. That said, only a handful of studies have evaluated EDCs commonly used in PCPs as it relates to race/ethnicity. These studies found differences in the pattern of use for hair and skin care products, cosmetics, nail polish, perfumes, and vaginal douches in non-pregnant populations [19, 30–34].

**Fig. 1:**
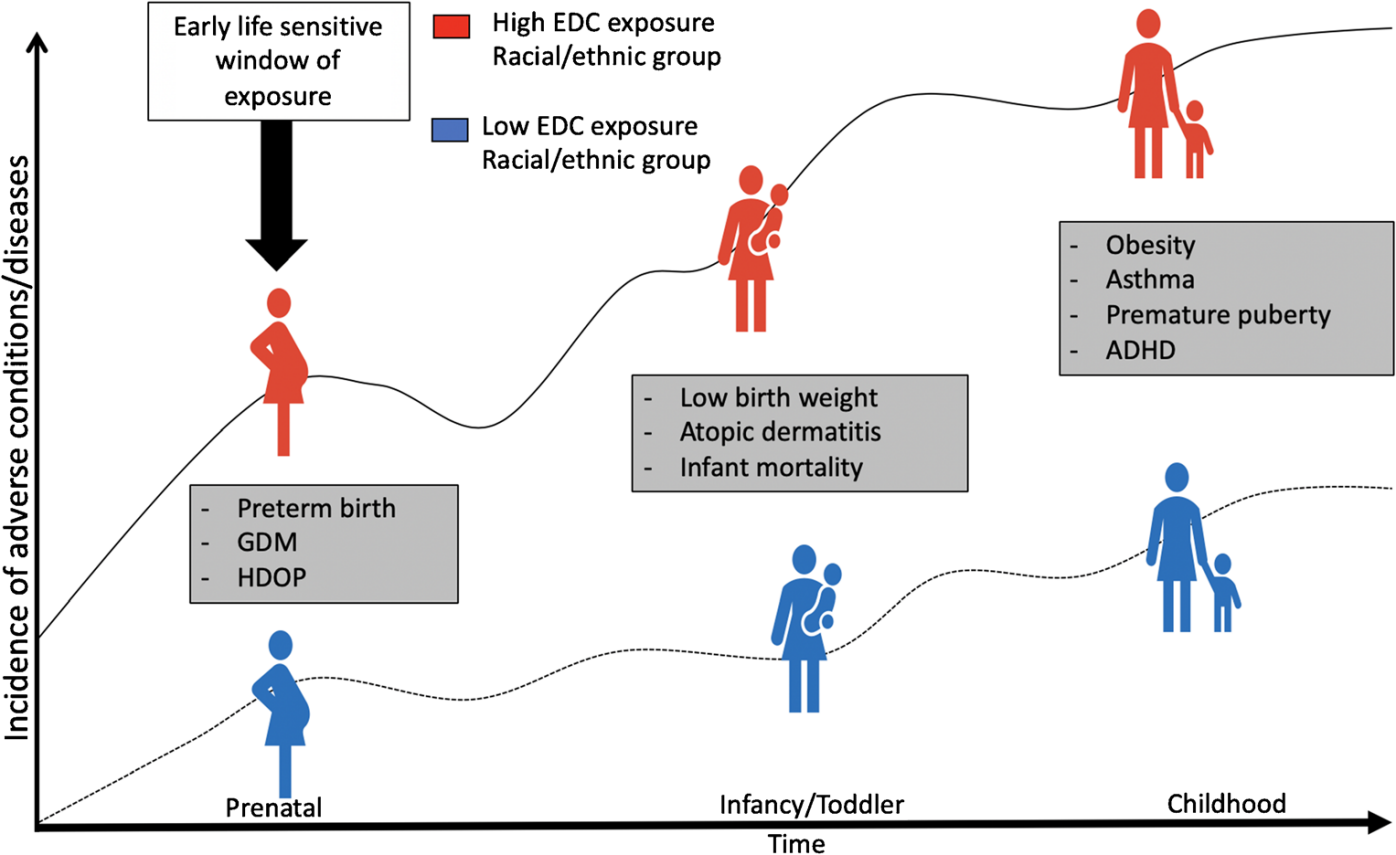
Examples of high and low cumulative EDC exposure and racial/ethnic disparities at each relevant pregnancy/prenatal period and childhood, showing the possible adverse health implications of the growing exposure disparity. ^*^GDM: gestational diabetes; HDOP: hypertensive disorders of pregnancy; ADHD: attention deficit hyperactivity disorder

Figure 1 conceptualizes the potential impact of heterogeneous levels of cumulative exposure across racial/ethnic groups throughout early life, showing the possible adverse health implications of the growing disparity. There is a need to investigate chemical concentrations and their sources within the same framework in health disparity research to identify the potential contribution of modifiable risk factors in persistent health disparities. As such, we conducted a scoping review to understand the current state of the literature on racial/ethnic differences in chemical exposures occurring during pregnancy and the prenatal period that are linked to potentially modifiable risk factors. For this, we summarized the literature that examines pregnancy and prenatal exposure to 8 chemicals or classes of chemicals previously identified as chemicals of concern found in PCPs (phthalates, parabens, benzophenone-3, triclosan, cyclic volatile methylsiloxanes, formaldehyde-releasing preservatives, 1,4-dioxane, and diethanolamine) [35–37] among women of different racial/ethnic backgrounds in the contiguous US and Puerto Rico. We also reviewed articles that report stratified data on pregnancy and prenatal exposure to the 8 chemicals or classes of concern, but do not examine the racial/ethnic disparities in exposure. Finally, we provide recommendations for research needed to fill the gap in the literature on EDC-associated personal care product use as an important contributor to racial/ethnic maternal and child environmental health disparities.

## Methods

Based on previous literature [35–37], we identified a total of 8 EDCs or chemicals of health concern found in personal care products. These included phthalates, benzophenone-3, parabens, triclosan, cyclic volatile methylsiloxanes, formaldehyde-releasing preservatives (formaldehyde, quaternium-15, DMDM hydantoin, imidazolidinyl urea, diazolidinyl urea, polyoxymethylene urea, sodium hydroxymethylglycinate, 2-bromo-2-nitropropane-1,3-diol (bromopol) and glyoxal), 1,4-dioxane, and diethanolamine. We excluded compounds such as asbestos and polycyclic aromatic hydrocarbons, which are not predominately identified as personal care product chemicals.

To conduct this scoping review on racial/ethnic disparities in pregnancy and prenatal exposure, studies were identified by searching PubMed/Medline (National Library of Medicine), EMBASE (Elsevier), Web of Science Core Collection, including the Science Citation Index and Conference Proceedings Citation Index- Science (Thomson Reuters), and the Cochrane Central Register of Controlled Trials (CENTRAL - cochranelibrary.com) from database inception through March 2020. Controlled vocabulary terms (i.e., MeSH, EMTREE) were included when available and appropriate. The search strategies were designed and executed by a librarian with expertise in scoping and systematic reviews (CM). Articles were included if the populations of interest were US-based and reported concentrations during pregnancy and the prenatal period of any of the 8 chemicals or classes of chemicals of interest by maternal race/ethnicity. No language limits or year restrictions were applied. See Supplemental Material 1 for details of the terms used for the search.

## Results

The search yielded 2491 references, resulting in 1539 unique records for screening. With the knowledge that there would be only a few studies that align with our aim of examining racial/ethnic disparities in pregnancy and prenatal exposure, we also included studies that reported urinary concentrations by racial/ethnic group. Specifically, we included studies that examined personal care product chemical concentrations during pregnancy and the prenatal period in racially/ethnically diverse cohorts or in racially/ethnically homogenous US minority populations (as a proxy for stratified data). We identified 3 studies that explicitly examined the racial/ethnic disparities in exposure to EDCs commonly used in personal care products during pregnancy and the prenatal period (tier 1) and 53 articles that presented racially/ethnically stratified exposure data in the contiguous US and Puerto Rico. For these 53 articles, given that identifying and evaluating differences in exposure by race/ethnicity was not the primary aim of the study, we classified these studies as tier 2.

Of the 56 articles identified as containing data on racial/ethnic differences in personal care product-associated EDC concentrations, all papers covered one or two chemical classes: phthalates (43 articles) or phenols (17 articles). Six papers reported on both chemical classes. Among the articles on phenols, most focused on triclosan and a few on benzophenone-3. No studies evaluating racial/ethnic differences in other chemicals, such as cyclic volatile methylsiloxanes and formaldehyde-releasing preservatives were found. Also, the vast majority of studies evaluated racially homogenous cohorts, specifically non-Hispanic Black and Hispanic populations.

### Phthalates

Phthalic acid esters are a class of industrial and multi-use chemicals that can be found in a variety of consumer products [37]. Phthalates are ubiquitous in our environment and can be detected in food, consumer, and personal care products, indoor air, indoor dust, and inside vehicles [38]. Due to their short half-life (from 5 h to less than 24 h), concentrations are representative of recent exposure compared to chronic or cumulative exposure [37, 39]. Unlike the parent compounds, it is thought that phthalate metabolites are bioactive, with the ability to bind to nuclear receptors, altering normal hormone functioning and the regulation of hormonal pathways [40–42]. With that, phthalate metabolites are typically measured to assess concentrations and potential exposures in populations.

Given their presence in personal care products, we focused on studies reporting information on low molecular weight phthalates, such as diethyl phthalate (DEP), di-iso-butyl phthalate (DiBP), and di-n-butyl phthalate (DBP) used in solvents, medications, and personal care products [38, 43]. Among those studies that assessed phthalates used in personal care products during pregnancy and the prenatal period, three US studies met the tier 1 criteria (Table 1) [44••, 45••, 46••]. Two studies that examined a population recruited from the Medical University of South Carolina found that non-Hispanic Black women had significantly higher urinary concentrations of MBP, mono-isobutyl phthalate (MiBP), monobenzyl phthalate (MBzP), and MEP compared to non-Hispanic White women (*p*<0.005) [44••, 45••]. Similarly, James-Todd et al. evaluated urinary phthalate metabolite concentrations in a diverse cohort and found higher concentrations of MBP, MiBP, MBzP, and MEP among non-Hispanic Black women, Hispanic women, and women who selected “Other” race/ethnicity compared to non-Hispanic White women. Specifically, non-Hispanic Black women had a baseline specific gravity adjusted geometric mean (GM) MEP of 286.0μg/L compared to 98.7μg/L for non-Hispanic White women. Interestingly, MEP decreased throughout pregnancy for Hispanic women and increased in late pregnancy for non-Hispanic Black women [46••]. Changes in metabolites may be attributed to different product use or changing perceptions of risk during pregnancy [47]. Importantly, these findings reflect higher exposure to personal care product phthalates, such as DBP and DEP during pregnancy, which may illustrate differences in modifiable product use by race/ethnicity.

**Table 1:** Tier 1 epidemiologic studies examining racial/ethnic differences in pregnancy/prenatal EDC exposure and disparities in health outcomes

**Author, Year**	**N (total and by race/ethnicity)**	**Cohort**	**Study Location**	**Chemical measured**	**Main findings**
(Bloom et al. 2019) [44••]	*n*=310; 152 Black, 158 White	N/A- Delivery at Medical University of South Carolina	Charleston, SC	-8 phthalate metabolites collected at two time points (T1 and T2 4-14 weeks later)	- Significantly greater concentrations of urinary MBP, MiBP, MBzP, MEHP, MEP, MMP, and ΣDBP among Black participants compared to White participants.. - Younger maternal age, lower income and educational attainment, and higher BMI were associated with greater urinary phthalate metabolites at T1 (*p* < 0.05). - Urinary phthalate metabolite concentrations were generally greater than those reported from a representative sample of women from NHANES (2011-2014).
(Wenzel, Brock, et al. 2018) [45••]	*n* =378; 186 African American, 192 Caucasian	N/A- Delivery at Medical University of South Carolina	Charleston, SC	-8 phthalate metabolites (single urine sample during the second trimester)	- Marital status (unmarried), lower income and educational attainment, higher BMI, and identifying as African American were associated with higher urinary phthalate metabolite concentrations. - Among African American participants, age, BMI, maternal education, and income were significantly associated with higher urinary phthalate metabolite concentrations. - Among White participants, marital status was significantly associated with higher urinary phthalate metabolite concentrations. - Urinary phthalate metabolite concentrations were similar to those reported from a representative sample of women from NHANES (2011-2012).
(James-Todd et al. 2017) [46••]	*n*= 350; 206 White, 55 Black, 19 Asian, 50 Hispanic, 20 Other	LIFECODES	Boston, MA	-9 phthalate metabolites collected at 4 time points (median 9.9, 17.9, 26.1, and 35.3 weeks gestation.)	- The highest urinary phthalate metabolite concentrations were found among Black and Hispanic participants throughout pregnancy. - MEP levels (specific gravity adjusted geometric mean) among Black participants were 286.0μg/L compared to 98.7μg/L for White participants. - MBzP levels among Hispanic participants (11.6 μg/L) and Black participants (10.2μg/L) were higher than among White participants (5.4 μg/L). -Among Hispanic participants, MEP levels were higher at the first time point but decreased later in pregnancy. In comparison, among Black participants, MEP levels increased later in pregnancy.

Evidence that product use may drive differences in low molecular weight phthalate exposure comes from a small set of studies. In a study of 186 pregnant non-Hispanic Black and Dominican women, perfume users had 2.3 times higher urinary MEP concentrations compared to non-perfume users [48]. These estimates were based on multivariable logistic regression parameters that were exponentiated to report the multiplicative fold change between users and non-users of perfume in the last 48 h. Another study in New York City found that non-Hispanic Black women were more likely to use all types of hair products, specifically products containing placenta or EDCs on the product labels—such as fragrance and parabens—compared to non-Hispanic White women [16].

Forty papers from nine cohorts met the tier 2 criteria of presenting racial/ethnic stratified phthalate exposure data, without an explicit focus on disparities (Table 2) [24, 48–86]. From those, 31 papers in three racially/ethnically homogenous US minority cohorts (Center for the Health Assessment of Mothers and Children of Salinas-CHAMACOS, Columbia Center for Children’s Environmental Health-CCCEH, and PROTECT) presented urinary concentrations of phthalate metabolites among pregnant Hispanic and non-Hispanic Black women. As some cohorts published multiple studies stratified by race/ethnicity, we present the most complete data in Table 2 from cohorts with multiple studies, in addition to those cohorts publishing single papers.

**Table 2:** Tier 2 epidemiologic studies (with the most complete data) reporting stratified concentrations of phthalate exposure during pregnancy/ the prenatal period

**Author, Year**	**Cohort**		**N (total and by race/ethnicity)**	**Exposure**	**Differences in exposure within cohort**	**Findings compared to a representative sample of women from NHANES**
(Morgenstern et al. 2017) [49]	CCCEH	Northern Manhattan and the Southern Bronx, NY	*n*=727; 473 Dominican, 254 African American	-8 phthalate metabolites measured from spot urine samples during the third trimester (33.8 ± 3.2 weeks gestation)	- MBzP concentrations significantly higher in African American women - MEHHP and MECPP concentrations were significantly higher in Dominican women	*No comparisons made*
(Hyland et al. 2019) [50]	CHAMACOS	Salinas Valley, CA	*n*=590 Mexican American	-11 phthalate metabolites measured in urine samples twice during pregnancy (median 13 and 26 weeks gestation)	*No comparison*	-Generally similar to those in NHANES around the same time period.
(Wenzel, Bloom, et al. 2018) [51]	Delivery at MUSC	Charleston, SC	*n*=380; 49.2% African American, 50.8% Caucasian	-8 phthalate metabolites measured in urine samples during the second trimester	- African American participants had significantly higher concentrations compared to White participants (*p*<0.05)	*No comparisons made*
(Mínguez-Alarcón et al. 2019) [52]	EARTH	Boston, MA	*n*=420; 350 White, 70 Black/Asian/Other	-8 phthalate metabolites measured once or twice per IVF cycle	-*No comparison*	*No comparisons made*
(Polinski et al. 2018) [53]	Healthy Start	Aurora, CO	*n*=446; 256 White, 108 Hispanic, 53 African American, 29 All Others	-15 phthalate metabolites from spot urine samples collected at 24-32 weeks gestation	- Lowest concentrations of ∑DEHP and ∑DBP among White women	-Compared to 2003–2004 NHANES, average concentrations of MBP, MBzP, MEP, MEHP, MEHHP, MEOHP and MECPP were lower in participants and MiBP concentrations were higher.
(Werner et al. 2015) [54]	HOME	Cincinnati, OH	*n*=34; 14 White,20 Nonwhite	-9 phthalate metabolites from spot urine samples measured at 16 and 26 weeks gestation	*No comparison*	*No comparisons made*
(Ferguson et al. 2015) [55]	LIFECODES	Boston, MA	*n*=482; 59% White, 16% Black, 26% Other	-9 phthalate metabolites from spot urine samples four times (10, 18, 26, and 35 weeks gestation) during pregnancy	-Higher concentrations of MBzP, MiBP, MEP, and MBP among African American or other race/ethnicity participants compared to White participants	-MEHP concentrations and DEHP metabolites were higher in African Americans. -Higher concentrations of MBzP, MiBP, and MEP among mothers who were African American (vs. White), had lower education levels, had public insurance (vs. private), and who had a higher BMI at visit 1.
(Ferguson et al. 2019) [24]	PROTECT	Northern Karst Region, Puerto Rico	*n*=1090	-19 phthalate metabolites or phthalate replacements from spot urine samples three times during pregnancy (20, 24, and 28 weeks gestation)	*No comparison*	-Concentrations of DBP and DiBP metabolites higher than general US population.
(Serrano et al. 2014) [56]	TIDES	Multi-site study	*n*=656; 460 White, 44 Asian, 83 Black, 45 Other, 24 More than one race	-8 phthalate metabolites+ sum DEHP metabolites from spot urine samples collected at each trimester	*No comparison*	-Comparable for all metabolites to the US populations of reproductive age.

When evaluating studies from relatively homogenous cohorts, we compared findings to data from the National Health and Nutrition Examination Survey (NHANES). For example, in the CHAMACOS study, a longitudinal birth cohort in Salinas Valley, California consisting of Mexican American women, pregnant women generally had phthalate metabolite concentrations that were consistent with trends in comparable US women from NHANES [50].

The CCCEH cohort comprised of non-Hispanic Black and Dominican pregnant women living in New York City reported higher GM concentrations of MnBP, MiBP, and MEP (approximately 33–34 weeks of gestation) compared to NHANES participants of similar ages [48, 60, 65, 67, 86]. Among Puerto Rican women participating in the PROTECT study aiming to evaluate the potential relationship between environmental chemicals and preterm birth in the Northern Karst Region, urinary phthalate metabolite concentrations that were collected three times during pregnancy (approximately 16–20 weeks, 20–24 weeks, and 24–28 weeks of gestation) had higher reported MEP and MBP concentrations compared to NHANES participants [24]. Additional studies with diverse participants reported similar or lower concentrations of phthalate metabolites compared to NHANES participants [51–56, 58, 79, 85].

Given that exposure during pregnancy and the prenatal period to phthalate metabolites has been associated with adverse maternal and child outcomes [13, 14, 57, 87, 88], it is imperative to consider the health effects of these higher exposures in racial/ethnic minorities. Future research is needed to understand whether these observed differences in exposure to phthalates associated with personal care products could contribute to some of the disparities seen in the phthalate-associated adverse health outcomes.

### Phenols

#### Triclosan

Triclosan (TCS) is an antibacterial agent that has been found in an array of personal care products, including hand soaps, hand sanitizers, toothpaste, and shampoos [89, 90]. With a short half-life of 21 h, exposure represents recent product use [91]. Differences in triclosan concentrations between countries and within the US have been attributed mainly to differential exposure sources and product use behaviors [92]. Triclosan has been shown to disrupt thyroid hormone homeostasis through activating the human pregnane X receptor (PXR) and the inhibition of diiodothyronine sulfotransferases [93, 94]. Animal studies have also demonstrated that triclosan may inhibit the circulation of hormones, resulting in altered gene expression in the placenta and impaired fetal development [95].

Fifteen tier 2 papers from eight different cohorts reported the concentrations of triclosan by race/ethnicity among pregnant women (Table 3) [28, 53, 57, 72, 96–106]. One study of Puerto Rican pregnant women reported higher concentrations of triclosan compared to a representative subset of women participating in NHANES [99]. Comparatively, several studies with predominantly non-Hispanic White participants from the Healthy Start study and the National Children’s Study (NCS) were found to have lower [53, 98] or similar triclosan concentrations compared to 2005–2010 NHANES [96, 97, 103].

**Table 3:** Tier 2-summary of epidemiologic studies (with the most complete data) that report stratified concentrations of phenols by race/ethnicity during pregnancy/ the prenatal period

**Author, Year**	**Cohort**		**N (total and by race/ethnicity)**	**Exposure**	**Differences in exposure within cohort**	**Findings compared to a representative sample of women from NHANES**
(Berger, Eskenazi, Balmes, et al. 2018) [71]	CHAMACOS	Salinas Valley, CA	*n*=392 Mexican American	-3 parabens (methyl-, propyl- and butyl paraben) -benzophenone-3, and -triclosan concentrations measured in urine samples collected twice during pregnancy (mean 13 and 26 weeks gestation)	*No comparison*	-Higher concentrations of methyl paraben compared to NHANES Mexican American women aged 18–45.
(Polinski et al. 2018) [53]	Healthy Start	Aurora, CO	*n*=446; 256 White, 108 Hispanic, 53 African American, 29 All Others	-4 parabens (methyl-, ethyl-, propyl-, and butyl paraben) -benzophenone-3, and -triclosan from spot urine samples collected at 24–32 weeks gestation	- Lowest concentrations of parabens among White women. -Highest concentrations of triclosan and benzophenone-3 among White women	-Compared to a 2005–2010 NHANES sample of pregnant woman aged 16–44 years, benzophenone-3 was higher, triclosan, methyl paraben and propyl paraben concentrations were similar.
(Mínguez-Alarcón et al. 2019) [52]	EARTH	Boston, MA	*n*=420; 350 White, 70 Black/Asian/Other	-2 parabens (methyl paraben and propyl paraben) measured once or twice per IVF cycle	*No comparison*	*No comparisons made*
(Etzel et al. 2017) [96]	HOME	Cincinnati, OH	*n*=378; 235 White, 117 Black, 26 Other	-Triclosan measured in urine samples twice during pregnancy (average 16 and 26.5 weeks gestation)	- Higher triclosan concentrations among White participants	-Triclosan concentrations consistent with NHANES.
(Aung et al. 2019) [97]	LIFECODES	Boston, MA	*n*=480; 282 White, 77 African American, 121 Other	-4 parabens (methyl, ethyl, propyl, and butyl parabens) and -benzophenone-3, measured in urine samples up to four times (median 9.7, 17.9, 26, and 35 weeks gestation) during pregnancy	- Higher benzophenone-3, butyl paraben, and ethyl paraben concentrations among White participants - Higher methyl paraben and propyl paraben concentrations among African American participants - Higher triclosan concentrations among other race/ethnicity participants	-Concentrations of urinary phenols and parabens mostly comparable to concentrations in pregnant women from the NHANES (2005–2010) and NCS (2009–2010). -Higher median concentrations of methyl and propyl parabens.
(Mortensen et al. 2014) [98]	National Children's Study (NCS)	US sample	*n*=506; 30 Black, 328 White, 99 Hispanic, 49 Other	-2 parabens (methyl paraben, propyl paraben) -benzophenone-3, and -triclosan, from third trimester urine samples	- Highest mean benzophenone-3 concentrations among non-Hispanic White participants - Highest mean triclosan concentration among Hispanic women	-Concentrations similar to those from pregnant women in NHANES 2005–2010 except triclosan (decline from 2005-2008).
(Ashrap et al. 2018) [99]	PROTECT	Northern Karst Region, Puerto Rico	*n*=1003	-4 parabens (ethyl-, methyl-, butyl-, and propyl parabens) -benzophenone-3, and -triclosan, from two spot urine samples at 16-20 and 24-28 weeks gestation	*No comparison*	-Phenol and paraben concentrations tended to be higher than levels measured in women of reproductive age from the general US population.
(Lee-Sarwar et al. 2018) [100]	Vitamin D Antenatal Asthma Reduction Trial (VDAART)	Boston, MA; St. Louis, MO; and San Diego, CA	*n*=467; 202 Black, 120 White, 109 Hispanic, 36 Other	-2 parabens (methyl paraben, and propyl paraben) -benzophenone-3, and -triclosan concentrations were quantified in maternal plasma samples pooled from the first and third trimesters	- Other race/ethnicity participants had higher reported triclosan, methyl paraben, and propyl paraben	-Maternal plasma concentrations of triclosan, methyl paraben, and propyl paraben differed significantly between maternal race/ethnicity groups. -Personal care product use frequency differed significantly by race/ethnicity for all personal care products except for leave-in conditioner.
(Pycke et al. 2014) [101]	N/A- Recruited from University Hospital of Brooklyn Prenatal Clinic	Brooklyn, NY	*n*=190; 81 African American, 78 Caribbean/West Indian, 12 African, 15 Latino/Hispanic, 4 Other	-Triclosan collected from random spot urine samples once during the sixth-ninth month of pregnancy	*No comparison*	-Compared to the general population of the US, substantially higher concentrations of triclosan and at a higher frequency.

When evaluating triclosan concentrations within a study by race/ethnicity, results were inconsistent. For example, data from the Healthy Start and the Health Outcomes and Measures of the Environment (HOME) study reported higher concentrations of triclosan among pregnant non-Hispanic White women compared to other racial/ethnic groups (participants were primarily non-Hispanic White) [53, 96]. On the other hand, two studies found that women with the race classified as “Other” had higher reported concentrations of triclosan [97, 100]. Larger multi-racial/ethnic studies are needed to further evaluate whether triclosan contributes to environmental health disparities, given these inconsistent findings.

#### Parabens

Parabens are another ubiquitous class of synthetic chemicals [107]. These chemicals are used as antimicrobial agents and preservatives for personal care products, pharmaceuticals, and food products to increase their shelf life. Methyl paraben, propyl paraben, and butyl paraben are the most commonly used parabens in personal care products [18, 108, 109]. This class of chemicals also has a short half-life (for example, butyl paraben was found to be excreted in 2.6±0.1 mg/24 h) [110], and biomarkers reflect more recent exposures [111]. Parabens are found to be weak estrogens that can bind to estrogen receptors α and β [112, 113]. Although less studied than phthalates or triclosan, US studies using NHANES have reported that Hispanic and non-Hispanic Black women across the US have higher concentrations of parabens compared to non-Hispanic White women [11, 114].

Thirteen tier 2 studies among 7 different cohorts reported racial/ethnic stratified concentrations of parabens among pregnant women (Table 3) [28, 52, 53, 57, 71, 97–100, 104–106, 115]. Compared to NHANES, one study of 1003 Puerto Rican women that collected urine at 16–20 and 24–28 weeks gestation found 2-fold higher median concentrations of butyl paraben [99]. Interestingly, like the PROTECT study, CHAMACOS also reported high concentrations of parabens compared to NHANES participants [71]. In a multi-ethnic study that explicitly reported concentrations of parabens for non-Hispanic Black and Hispanic/Caribbean immigrants in the US, relative to a representative sample of women participating in NHANES the median concentrations of methyl paraben and propyl paraben were 4.4-fold and 8.7-fold higher, respectively [115]. These findings suggest further investigation as to whether higher paraben concentrations could contribute to related health disparities.

#### Benzophenone-3

Benzophenone-3 (BP3) is a UV-filter found in sunscreens and other personal care products [105, 116]. BP3 is ubiquitous among individuals living in the US indicating widespread exposure [117]. Benzophenones are found to cause androgenic effects and disrupt nuclear and estrogen receptors [118]. Specifically, BP3 is found to activate estrogen receptors α and β in animal studies [119]. Temporal trends from the US NHANES population indicate that non-Hispanic Blacks have lower and stable mean concentrations of BP3, while non-Hispanic Whites have higher concentrations [120]. This finding may be explained by the evidence that non-Hispanic Blacks are seven times less likely to use sunscreen compared to non-Hispanic Whites who report severe sunburns [121].

Nine studies in five different cohorts met the tier 2 criteria and reported racial/ethnic stratified concentrations of BP3 metabolites among pregnant women (Table 3) [28, 49, 65, 75, 99–103]. Data from the PROTECT study reported higher concentrations of BP3 compared to women of reproductive age from NHANES [99]. Another study using the Healthy Start cohort—with a diverse, but predominantly non-Hispanic White study population—reported higher BP3 concentrations compared to a sample of pregnant NHANES participants [53]. On the other hand, the CHAMACOS cohort participants had similar BP3 concentrations to women participating in NHANES [71]. Within the LIFECODES pregnancy cohort, non-Hispanic White women had higher concentrations of BP3 compared to non-Hispanic Black women and women identified as “Other” [97]. Future studies should assess the impact of these differences on associated health outcomes to determine whether this exposure contributes to protective or adverse health effects.

### Cyclic Volatile Methylsiloxanes, Formaldehyde-Releasing Preservatives, 1,4-Dioxane, and Diethanolamine

To our knowledge, no US-based studies have examined the racial/ethnic differences in pregnancy and prenatal exposure to cyclic volatile methylsiloxanes, formaldehyde-releasing preservatives, 1,4-dioxane, or diethanolamine. There is limited epidemiologic research, in general, examining exposure to the aforementioned chemicals as they relate to exposure differences by race/ethnicity, as well as health outcomes, leaving a gap in the knowledge regarding personal care product chemicals of concern.

While epidemiological studies are limited for these chemicals, research has begun to explore the potential mechanisms of action, particularly for cyclic volatile methylsiloxanes and diethanolamine as potential EDCs. Specifically, research has examined chemicals in products used and marketed to non-Hispanic Black women. For example, Helm et al. tested 18 products used by non-Hispanic Black women and found that a hair relaxer and multiple anti-frizz products had concentrations of octamethylcyclotetrasiloxane (D4), decamethylcyclopentaasiloxane (D5), and dodecamethylcyclo-hexasiloxane (D6) above 1000 μg/g [36]. Additionally, diethanolamine was found in a leave-in conditioner and multiple hair relaxers at concentrations between 100–1000 μg/g. Expanding research aims to include other emerging EDCs of concern that may have racial/ethnic differences in exposure, particularly given the racial/ethnic differences in product use [16, 19], is needed to help further explain the disparities in maternal and child health outcomes.

## Call to Action for Further Research

In this review, we presented evidence on exposure disparities to EDCs associated with personal care products—phthalates and several phenolic compounds (triclosan, parabens, and benzophenone-3)—during pregnancy and the prenatal period. We aimed to provide a summary of the evidence on racial/ethnic differences in exposure to support the argument that disparities in exposure to these EDCs during the pregnancy and prenatal period could contribute to the disparities in maternal and child health outcomes that disproportionately burden certain racial/ethnic groups. Since product use is modifiable, fully examining pregnancy and prenatal exposures to EDCs commonly used in personal care products could provide an important opportunity for future intervention and public health recommendations.

While 43 studies reported phthalate metabolite concentrations by race/ethnicity, few studies reported stratified concentrations of other personal care product chemicals and even less aimed to examine disparities in exposure, particularly as it related to health outcomes. In total, only three articles’ primary objective was to examine the racial/ethnic disparities in exposure during pregnancy and the prenatal period. Instead, the majority of studies simply presented stratified exposure data by race/ethnicity as a descriptor of their exposure of interest. Several potentially important chemicals remained missing, and importantly, little information was available on Asian or ‘Other’ racial/ethnic groups. An important example is that emerging evidence suggests that triclocarban may be an important chemical to evaluate [99, 101, 104–106]. While not originally covered in this review, Puerto Rican women were found to have 37-times higher levels of triclocarbon than women of reproductive age participating in NHANES [104]. Reasons for these differences are unclear and few studies have evaluated sources of exposure differences, including sociocultural drivers of usage patterns that could contribute to these disparities.

As a result, we suggest utilizing both the information gained from studies describing racial/ethnic differences and those describing associations between EDCs and adverse health outcomes to fill the gaps in the research examining environmental racial/ethnic disparities, particularly as it relates to pregnancy and prenatal exposure to EDC-associated personal care products. To leverage this information and reduce health disparities in this area, we suggest:

- Increased research examining endocrine disrupting chemicals as a potential risk factor for racial/ethnic disparities in maternal and child health outcomes among large, diverse populations either as single studies or in pooled studies.
- Examination of personal care product chemical exposure in racially/ethnically diverse populations/cohorts, including detailed reporting of race/ethnicity to examine potential disparities in exposure among women and children in other racial/ethnic groups, including Asian, and mixed or multiracial participants.
- Assessment of personal care products and other sources of EDCs in the same studies to understand associations and develop interventions for these potentially modifiable risk factors of racial/ethnic disparities in EDC exposures and related health disparities [122].
- Joint examination of sociocultural and socioeconomic determinants of disparities in exposure and race/ethnicity, as they relate to EDC and health associations
- Inclusion of results stratified by race/ethnicity when reporting endocrine disrupting chemical concentrations
- Examination of pregnancy and prenatal exposure to personal care product chemicals that have not been studied, but may be particularly important to vulnerable populations based on patterns of personal care product use [36] (ex: cyclic volatile methylsiloxanes, formaldehyde releasing preservatives, 1,4-dioxane, diethanolamine)

## Conclusion

Based on the current literature, the overall pattern of exposure to environmental EDCs commonly found in personal care products differs by race/ethnicity during pregnancy and the prenatal period. As this is a critical time period of development for a variety of health outcomes ranging from pregnancy complications to asthma and neurobehavioral outcomes in children, understanding all contributors to disparities linked to these conditions is imperative. While the vast majority of studies simply adjusted for race/ethnicity, a number of studies—mainly those focused on phthalates and phenols—provided data on chemical concentrations stratified by race/ethnicity or within racial/ethnic US minority-only populations, revealing that non-Hispanic Blacks and Hispanics were high-exposure groups for many of these EDCs. Given that these same groups are also at higher risk of many of the associated health outcomes linked to these EDCs, it is critical to better understand whether patterns of personal care product use can explain these disparities. This review calls for future work to better elucidate the contribution that personal care product EDCs may have to existing racial/ethnic disparities in both exposure and health outcomes. Studies are needed to understand the sociocultural drivers of these exposure disparities, and future work must consider possible interventions to reduce exposure and persistent racial/ethnic health disparities related to these chemical exposures.

## Supplementary Information

ESM 1 (DOCX 16 kb)
