# Supplementary material for: Racial/Ethnic Disparities in Pregnancy and Prenatal Exposure to Endocrine-Disrupting Chemicals Commonly Used in Personal Care Products
Source: Curr Environ Health Rep. 2021 May 27;8(2):98–112. doi: 10.1007/s40572-021-00317-5 (PMC8208930; doi:10.1007/s40572-021-00317-5)
Supplement: Supplementary file 1 — (DOCX 16 kb) [file 40572_2021_317_MOESM1_ESM.docx]

**Supplemental Material:**

Supplemental material 1: SEARCH STRATEGIES

PUBMED (NLM):

"Prenatal Exposure Delayed Effects"[Mesh] OR "Maternal Exposure"[Mesh] OR "Maternal-Fetal Exchange"[Mesh] OR "Gestational Age"[Mesh] OR antenatal[tiab] OR gestation age[tiab] OR gestational age[tiab] OR in-utero[tiab] OR maternal exposure[tiab] OR maternal fetal[tiab] OR pregnancies[tiab] OR pregnancy[tiab] OR pregnant[tiab] OR prenatal[tiab] OR transplacental[tiab]

AND

"Phthalic Acids"[Mesh] OR "phthalic acid" [Supplementary Concept] OR "diethyl phthalate" [Supplementary Concept] OR "Benzophenones"[Mesh] OR "benzophenone" [Supplementary Concept] OR "oxybenzone" [Supplementary Concept] OR "Parabens"[Mesh] OR "Triclosan"[Mesh] OR "Siloxanes"[Mesh] OR "Formaldehyde"[Mesh] OR "quaternium-15" [Supplementary Concept] OR "1,3-dimethylol-5,5-dimethylhydantoin" [Supplementary Concept] OR "imidazolidinyl urea" [Supplementary Concept] OR "diazolidinylurea" [Supplementary Concept] OR "monomethylolglycine" [Supplementary Concept] OR "bronopol" [Supplementary Concept] OR "Glyoxal"[Mesh] OR "Dioxanes"[Mesh] OR "1,4-dioxane" [Supplementary Concept] OR "diethanolamine" [Supplementary Concept] OR 2-bromo-2-nitropropane-1,3-diol [tiab] OR benzophenone*[tiab] OR bronopol[tiab] OR bronosol[tiab] OR butylparaben*[tiab] OR cyclopentasiloxane[tiab] OR cyclotetrasiloxane[tiab] OR diazolidinyl urea[tiab] OR diazolidinylurea[tiab] OR diethanolamine[tiab] OR diethylphthalate*[tiab] OR diolamine[tiab] OR dioxan[tiab] OR dioxane[tiab] OR ethanedial[tiab] OR ethanedione[tiab] OR formaldehyde[tiab] OR glyoxal[tiab] OR hydroxybenzoic acid[tiab] OR imidazolidinyl urea[tiab] OR imidazolidinylurea[tiab] OR irgasan[tiab] OR organosiloxane*[tiab] OR oxybenzone*[tiab] OR methylparaben*[tiab] OR methylsiloxane*[tiab] OR paraben[tiab] OR parabens[tiab] OR phthalate*[tiab] OR phthalic acid*[tiab] OR propylparaben*[tiab] OR quaternium-15[tiab] OR siloxan[tiab] OR siloxane*[tiab] OR silicone*[tiab] OR sodium hydroxymethylglycinate[tiab] OR triclosan[tiab])

AND

"blood" [Subheading] OR "urine" [Subheading] OR "Serum"[Mesh] OR blood[tiab]OR serum[tiab] OR urine[tiab] OR urinary[tiab]

NOT ("Animals"[Mesh] NOT "Humans"[Mesh])

- 728 results 03/17/2020

EMBASE (ELSEVIER / embase-com):

Advanced search settings:

- mapping features turned of

source: Embase

1)

'prenatal exposure'/de OR 'maternal exposure'/de OR 'gestational age'/de

2)

(antenatal OR 'gestation age' OR 'gestational age' OR 'in utero' OR 'maternal exposure' OR 'maternal fetal' OR pregnancies OR pregnancy OR pregnant OR prenatal OR transplacental):ab,ti

3)

('phthalic acid derivative'/de OR 'phthalic acid'/de OR 'phthalic acid diethyl ester'/de OR 'benzophenone derivative'/de OR 'benzophenone'/de OR oxybenzone OR '4 hydroxybenzoic acid ester'/de OR 'triclosan'/de OR 'siloxane'/de OR 'formaldehyde'/de OR 'quaternium 15'/de OR 'diazolidinyl urea'/de OR 'bronopol'/de OR 'glyoxal'/de OR 'dioxane'/de OR 'diethanolamine'/de) NOT ([animals]/lim NOT [humans]/lim)

4)

('2-bromo-2-nitropropane-1,3-diol' OR benzophenone* OR bronopol OR bronosol OR butylparaben* OR cyclopentasiloxane OR cyclotetrasiloxane OR 'diazolidinyl urea' OR diazolidinylurea OR diethanolamine OR diethylphthalate* OR diolamine OR dioxan OR dioxane OR ethanedial OR ethanedione OR formaldehyde OR glyoxal OR 'hydroxybenzoic acid' OR 'imidazolidinyl urea' OR imidazolidinylurea OR irgasan OR organosiloxane* OR oxybenzone* OR methylparaben* OR methylsiloxane* OR paraben OR parabens OR phthalate* OR 'phthalic acid*' OR propylparaben* OR 'quaternium-15' OR siloxan OR siloxane* OR silicone* OR 'sodium hydroxymethylglycinate' OR triclosan) NOT ([animals]/lim NOT [humans]/lim)

5)

'blood level'/de OR 'maternal serum'/de OR 'urine level'/de OR 'urinalysis'/exp

6)

(blood OR serum OR urine OR urinary):ab,ti

(1 OR 2) AND (3 OR 4) AND (5 OR 6)

- 884 RESULTS 03/17/2020

COCHRANE CENTRAL REGISTER OF CONTROLLED TRIALS (COCHRANE LIBRARY, WILEY):

antenatal OR "gestation age" OR "gestational age" OR "in utero" OR "maternal exposure" OR "maternal fetal" OR pregnancies OR pregnancy OR pregnant OR prenatal OR transplacental

AND

"2-bromo-2-nitropropane-1,3-diol" OR benzophenone* OR bronopol OR bronosol OR butylparaben* OR cyclopentasiloxane OR cyclotetrasiloxane OR "diazolidinyl urea" OR diazolidinylurea OR diethanolamine OR diethylphthalate* OR diolamine OR dioxan OR dioxane OR ethanedial OR ethanedione OR formaldehyde OR glyoxal OR "hydroxybenzoic acid" OR "imidazolidinyl urea" OR imidazolidinylurea OR irgasan OR organosiloxane* OR oxybenzone* OR methylparaben* OR methylsiloxane* OR paraben OR parabens OR phthalate* OR "phthalic acid*" OR propylparaben* OR "quaternium-15" OR siloxan OR siloxane* OR silicone* OR "sodium hydroxymethylglycinate" OR triclosan

AND

blood OR serum OR urine OR urinary

- 47 trials 03/17/2020

- 24 unique references after removing duplicates

WEB OF SCIENCE (THOMSON REUTERS):

segments searched:

Science Citation Index + Conference Proceedings Citation Index- Science

In TITLE only:

"2-bromo-2-nitropropane-1,3-diol" OR benzophenone* OR bronopol OR bronosol OR butylparaben* OR cyclopentasiloxane OR cyclotetrasiloxane OR "diazolidinyl urea" OR diazolidinylurea OR diethanolamine OR diethylphthalate* OR diolamine OR dioxan OR dioxane OR ethanedial OR ethanedione OR formaldehyde OR glyoxal OR "hydroxybenzoic acid" OR "imidazolidinyl urea" OR imidazolidinylurea OR irgasan OR organosiloxane* OR oxybenzone* OR methylparaben* OR methylsiloxane* OR paraben OR parabens OR phthalate* OR "phthalic acid*" OR propylparaben* OR "quaternium-15" OR siloxan OR siloxane* OR silicone* OR "sodium hydroxymethylglycinate" OR triclosan

AND

in TOPIC:

antenatal OR "gestation age" OR "gestational age" OR "in utero" OR "maternal exposure" OR "maternal fetal" OR pregnancies OR pregnancy OR pregnant OR prenatal OR transplacental

AND

in TOPIC:

blood OR serum OR urine OR urinary

- 832 results 03/17/2020

- 387 unique results after removing duplicates

TOTAL REFERENCES RETREIVED ACROSS ALL DATABASES: 2,491

- 1539 unique references in Covidence project for screening
